# The role of the household in the social inclusion of children with special needs in Uganda – a photovoice study

**DOI:** 10.1186/s12887-021-02805-x

**Published:** 2021-09-06

**Authors:** Caroline Masquillier, Sara De Bruyn, David Musoke

**Affiliations:** 1 Department of Sociology, University of Antwerp, Antwerp, Belgium; 2 School of Public Health, Makerere University, Kampala, Uganda

**Keywords:** Social Inclusion, Children with Special Needs, Household, Photovoice Study, Uganda

## Abstract

**Background:**

Social inclusion establishes a basis for the overall wellbeing of children with special needs. Although children’s lives are centred around the household, little is known about the household’s influence on social inclusion. Therefore, the aim is to investigate the household’s role in the social inclusion of children with special needs in Uganda.

**Methods:**

Twelve carers of children with special needs participated in this photovoice study on the outskirts of Kampala, Uganda – including a training workshop, home visits, in-depth individual interviews and focus group discussion.

**Results:**

The social inclusion of children with special needs is highly complex because it has the potential to both benefit and cause harm. The results show that when a disability is socially devalued to a certain degree, carers and their household members have to deal with the ongoing process of stigma management. Depending on the characteristics of the child, carer and household, this can lead to an upward spiral towards visibility or a downward spiral towards concealment – reinforcing social inclusion or stigma, respectively.

**Conclusions:**

Despite the fact that there is disability among Ugandan children it remains a ‘hidden reality’. This research helps to reveal this hidden reality by understanding the role of the household in social inclusion in a stigmatized context.

## Introduction

Approximately 13 % of Ugandan children live with some form of disability [1]. In order to ensure the protection of these 2.5 million children from violence [2–4], poverty [2] and poor access to healthcare [2, 3], international organizations such as UNICEF have emphasized that it is indispensable to focus on their social inclusion in education, rehabilitation, cultural and recreational activities [4, 5]. Social inclusion is important for children living with a disability, as it promotes happiness, self-esteem, confidence and mental health [2, 6]. As Hall (2009) noted in her qualitative meta-analysis, it is important for children with a disability to gain ‘a sense of connectedness and belonging, to grow and develop in their abilities, and to procure a sense of satisfaction in their lives’ ([7]: p. 172). Social inclusion, therefore, establishes a basis for the overall wellbeing of a child living with a disability [2, 8, 9].

There are a myriad of definitions of what constitutes social inclusion for children living with a disability, resulting in opacity in meaning and a range of interpretations [2, 10–12]. The definition of social inclusion has often been influenced by theoretical perspectives on disability [12]. According to the bio-psychosocial model, for example, disability is seen as both a medical diagnosis and a social construction, involving interaction between the child’s health and environmental factors [9, 13]. This view is also reflected in the definition proposed by the World Health Organization, which defines disability as ‘a dynamic interaction between health conditions and contextual factors, both personal and environmental’ ([14]: p.4). In line with the current view on disability, this article adopts the definition of Cobigo et al. [10], for whom social inclusion is ‘the result of complex interactions between personal and environmental factors which increases an individual’s opportunities to contribute to society’ ([10]: p.81).

Foregrounding the importance of such environmental factors, Simplican et al. (2015) identified interpersonal relationships and community participation as the two life domains that are vital for social inclusion [2], while Chenoweth et al. (2004) focused on the role of social capital in social inclusion [15] and Murray et al. (2006) drew attention to the importance of the social context and relationships in the lives of children with a disability [16]. At the interpersonal level, the role of households is increasingly recognized to be important for social inclusion [17], as the majority of people living with disability live in a household or family^1^ [2, 17, 22]. The household constitutes the essential unit of social organization in daily life, negotiating the social, economic and cultural meanings of disability [11]. Furthermore, previous research has shown that the majority of the social relationships of individuals living with a disability are mediated through their households and families [17]. The interrelatedness and interdependence of a child living with a disability and their household context should thus be taken into account in research on social inclusion [13, 23–25]. In recent years, researchers have started to focus on the importance of this household level for social inclusion, with studies conducted in India [11], the United States [26] and Australia [15]. However, to date, ‘research is needed into the physical, cultural, social and economic barriers which impede the inclusion of disabled children in African countries’ ([4]: p. 9). To fully disentangle the complex patterns of inclusion and its barriers, this study aimed to investigate the role of the household in the social inclusion of children with special needs in a Ugandan setting.

## Methodology

As the research was to be undertaken in collaboration with children with special needs and their carers [4], photovoice was chosen to answer the research questions [27]. In this method, carers themselves produce visual representations (using cameras) of aspects of their children’s life related to social inclusion and its barriers. These images are then used as visual stimuli in subsequent in-depth individual qualitative interviews and focus group discussions with the carers [28–31].

### Study setting

Participants were recruited from the ‘parent’s support group’ of a local non-governmental organization (NGO) which aims to improve the quality of life of children with a disability by promoting and protecting their rights. The NGO is located in Nansana division, Wakiso district, which is a densely populated area consisting predominantly of informal dwellings on the outskirts of Kampala (Uganda’s capital city) in the central region of the country.

### Study design

After giving their written informed consent, the carers participated in a training workshop, which provided them with the knowledge and skills required for the research, such as a detailed explanation about the research process, camera use, use of photography in research, and ethics in research and photography [32, 33]. The carers included mothers and fathers, as well as aunts and grandmothers, who all took part in the workshop. The participants also received low-budget cameras for use during the research project and were asked to document over two weeks the ways in which their children with a disability were included in education, rehabilitation, cultural and recreational activities. Furthermore, the carers were asked to also take photos which identified and reflected on the barriers that impeded their children’s social inclusion.

After the respondents generated images related to the research question, the pictures were used as visual stimuli during in-depth interviews aiming to elicit more information about what the pictures depicted and why they had been taken [34]. Following the SHOWeD guidelines, the photographs were discussed in person with each of the carers [27]. The method is participatory in the sense that the respondents themselves chose which pictures to take and which images were prioritized for discussion and joint analysis in the in-depth interview [27]. The respondents also provided captions for each of the five pictures they selected as representing a response to the research question. Every respondent presented these five pictures to the other respondents in a closing focus group discussion. After the presentation of every respondent’s pictures, the focus group discussion offered the carers a platform to discuss the research questions and share their own experiences and viewpoints.

The interviews and focus group discussion were conducted in the preferred language of the interviewees (which was either English or the local language – Luganda), with the assistance of a local female Ugandan researcher. Interviews and the focus group discussion were audio-recorded, transcribed verbatim and translated into English (for those conducted in Luganda). Transcripts and observatory notes were imported into NVivo version 12 for analysis.

### Data analysis

The verbal clarifications and responses to visual stimuli (i.e. the pictures taken by the respondents) were analysed by the principal investigator. A sample of the transcripts was analysed by another researcher. Codes were compared with this researcher’s codes and similarities and differences discussed. The data was analysed carefully by reading and rereading the transcripts of the in-depth interviews and focus group discussion in accordance with the abductive analysis procedures described by Timmermans and Tavory [35].

### Ethical considerations

Information about the study, its purpose and design was explained in an easily understandable way to the participants. This information was also distributed by means of an information letter to the participants. Subsequently, informed written consent was obtained from the participants before study enrolment. Participants were informed that they could withdraw from the study at any time. The study was approved by the Ethics Committee of Social and Human Sciences, University of Antwerp (SHW_17_04_03).

To minimize the potential risks to participants, the research process was developed with specific attention paid to the unintended social effects on the actual and future wellbeing of those involved. For example, the carers were given time to reflect on their decision to participate and informed consent was sought at several moments in the research process. Furthermore, during the training workshop, several ethical aspects were discussed, such as how to approach and inform community members about the photovoice initiative, and how to seek consent before taking pictures [27, 33]. No photographs of individuals taken for the research were released or used in any form without the consent of both the photographer and those photographed [27, 33]. The names used in the quotes are pseudonyms; no original names have been used in this article.

## Results

We will first describe the results related to living with a child with special needs in the context of misconceptions and stigma. Second, the way in which stigma in the setting studied influences the social inclusion of the child with special needs will be outlined – by describing an upward spiral towards social inclusion or a downward spiral towards concealment. Finally, we will discuss the influence of individual characteristics of the child and carer, the household and the social network of the household on this social inclusion process.

### Living with a child with special needs in a context of misconceptions and stigma

While searching for the right diagnosis of their child’s disability, many carers were confronted by the limits of the Ugandan healthcare system. Respondents indicated that often doctors were not able to identify the disability that their child had. Various carers had to visit several doctors before they received the correct diagnosis. Moreover, even if their child received a correct diagnosis, sufficient healthcare or adapted schooling were rarely available. The absence of specialized services exemplified the lack of awareness about disability in Ugandan society.

*We have so many special needs but we don’t have doctors who know special needs children and that’s why even it is hard to tell from the start that the child has a special need. They [doctors] are not aware.* (Mother of Mukisa)

Misconceptions thrive within the context of a lack of knowledge and awareness about disability. Our research findings indicated that there was a belief in the Ugandan community studied that mothers who give birth to a child with special needs have been cursed or are being punished by God. Others think the child’s carers must have sacrificed the child’s health for other positive life outcomes such as becoming wealthy. Respondents indicated that some people thought a child with disabilities was bewitched or contagious, so that caring for or breastfeeding a child with special needs might delay the mother’s next pregnancy.

*We are ignorant about special needs, most of us, most of the parents, most of the people in Uganda, they are ignorant about special needs. They see special needs children as a curse, as if you have done wrong to God, as if you have sacrificed for the riches, for the parents to get the riches and which is not true. So, that’s ignorance.* (Mother of Sanyu)

The results showed that a lack of awareness and misconceptions about disability reinforced stigma. In a context where ignorance about special needs thrives, the carers indicated that they were confronted with various expressions of stigma. This was sometimes expressed in subtle ways, such as others getting out of a shared taxi. However, we also found that stigma was often expressed in very overt ways. Carers of children with special needs frequently experienced a negative response from their community, neighbours or family, who often saw the children as a waste of time and money. Some children were excluded from activities, others were confronted with violent behaviour, while some carers even faced exclusion from their families.

*All the time Dembe would want to play with the children, with her [another woman’s] children, but she could just ignore Dembe and just push her: ‘You go back to your house we don’t want to see you’. […] Then they could beat her from there. So that woman never liked Dembe. Actually, she is the one who made me move from that place to come here. Because I never wanted to fight with her, so I decided to move and leave that place and leave her. Because she never liked Dembe.* (Mother of Dembe)

The effects of stigmatization were numerous. Respondents indicated that the emotional impact of being faced with stigmatizing responses and behaviour cannot be underestimated – both for the child and the carer. Some carers were forced to move to another neighbourhood, while others tried to hide their child. In some extreme cases, carers or family members even thought about killing their child with special needs.

*Before I bought a car, I think Ojore was one of those people who pushed me to buy a car. Not because I was wealthy or I had had it, you get? Every day in a taxi then he cries, then he makes noise and people begin to look at you in a funny way. And, of course, looking at how he behaves, then I overheard a lady, [it] was like, my God, instead of having such a child, he rather dies. If you hear it and you are a mother, you are heartbroken.* (Mother of Ojore)

*They [extended family] wanted me to kill my child after just one week. This kid who does not have arms and legs.* (Father of Kigongo)

### Social inclusion in a stigmatized context

**Fig. 1:**
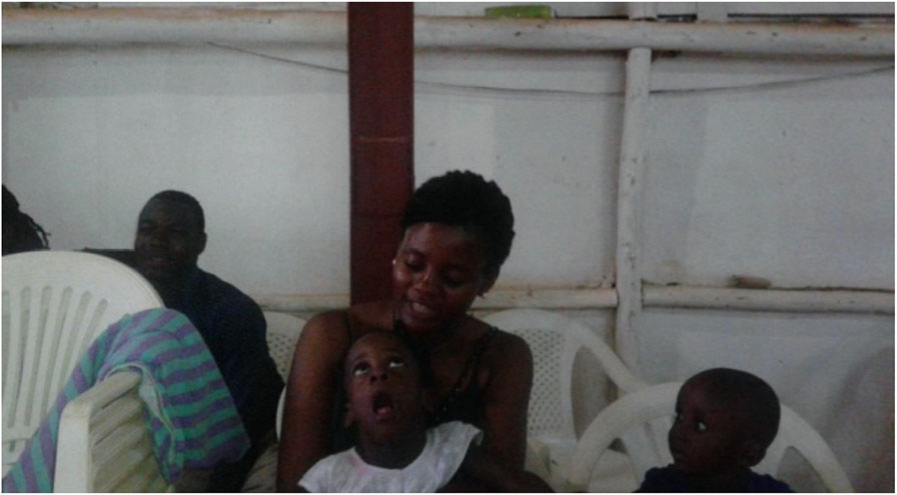
Source: Father of Namono

Carers of children with special needs defined social inclusion as having their children involved in daily activities, such as household chores, play and going to school. When discussing the concept of social inclusion, the carers emphasized the importance of their children being free to interact with others, with or without a disability, within the family and community. In accordance with the carers’ understanding of social inclusion, children in this study were socially included by participating in household chores, such as collecting water, washing dishes, washing clothes and playing games with siblings. In addition, children with special needs took part in social activities outside the household, especially in the church, which seemed to be one of the most important places in which the social inclusion of children with special needs and their household members could occur – as shown in Fig. 1.

*We were at church, Namono was with her friends. When she saw the friends, she would get excited to see other children in Sunday school.* (Mother of Namono)

**Fig. 2:**
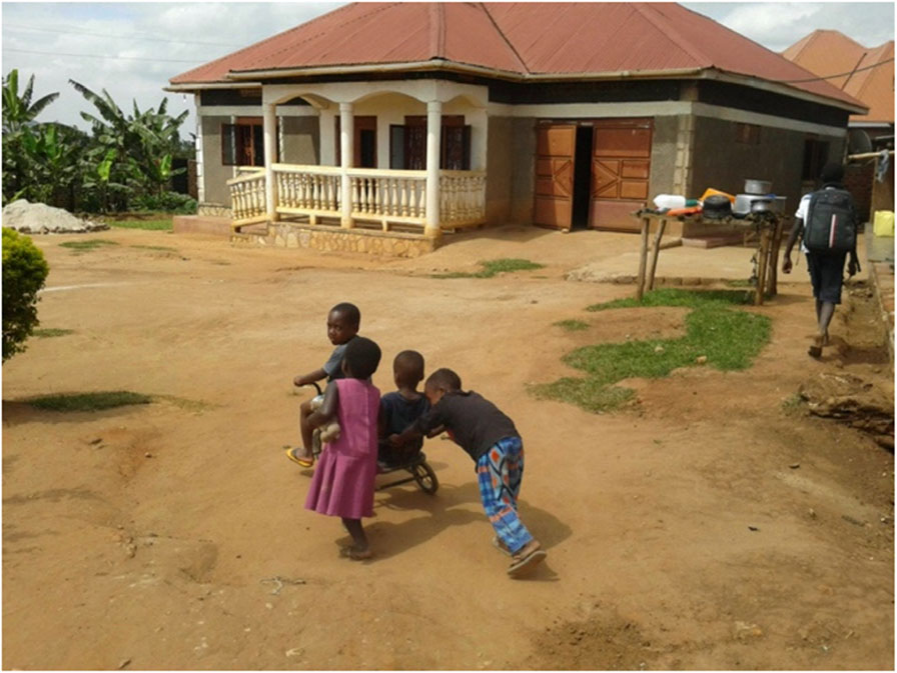
Source: Mother of Mukisa

Furthermore, most of the children with special needs played with other children in the neighbourhood, as presented in Fig. 2. Moreover, three children who were part of the study went to an inclusive school where they were in a classroom with children without a disability. The carers indicated that these various forms of social inclusion were important for several reasons, such as improving their child’s sense of belonging and their abilities, building confidence, making them feel that they are not alone but loved, and stimulating their development.

*The picture I normally take is when Mukisa is at home with his friends, he has a bicycle and whenever he is using his bicycle, his friends usually love it a lot and so they are always together and always calling out to him and so they are always playing together and it’s always making me happy.* (Mother of Mukisa)

Because there is a certain degree to which disability is socially devalued in the Ugandan community, the carers and their household members had to deal with an ongoing process of stigma management. In such a context, as the results show, social inclusion is a process rather than an outcome. The carers indicated that a constant effort was required to facilitate the inclusion of their children.

*They used to see her badly. They used to see her badly. Because one day it annoyed me when we entered in church and she started crying, she started disturbing [others] and the pastor’s wife said: ‘You take that child outside!’ […] I explained to them that Abbo is sick. They started learning her behaviours.* (Grandmother of Abbo)

*The most important thing as I have said is to change the attitude of teachers. My greatest worry with the teachers was they beat him up thinking he is just stubborn. Calling him dumb, stupid, that he doesn’t understand. So, that’s why before I took him to an inclusive school, I had to make awareness. (Mother of Akiki)*

The carers had to constantly find a balance between social inclusion and a potential exposure to negative responses and stigma. In such a context, socially including a child with special needs may be highly complex for carers and their household. It has the potential to both benefit and cause harm. On the one hand, social inclusion may offer the promise of additional social support for the child. On the other hand, social inclusion might make the carers and the child more vulnerable to stigma. We found that an upward spiral towards visibility in the community might occur, resulting in more social inclusion, but a downward spiral towards concealment might also occur, which reinforces stigma. We should also note that in this study these upward or downward spirals were identified in the close community of the household, namely the church, neighbours and extended family.

#### Upward spiral towards visibility

The research findings indicate that talking more openly about disability in the community stimulates awareness, which in turn might fight stigma. This upward spiral towards visibility facilitates social inclusion. We found that various carers took up the role of being advocates to create awareness about special needs. The respondents indicated that talking openly about disability in the Ugandan setting that we studied was not easy because of the many misconceptions. Some respondents indicated that they also attempted to identify other children with special needs in their community and provide the information they could. If they could not answer other carers’ questions, they referred them to the NGO, where their own children were taking part in the daycare programme. As mentioned above, the church played a central role in the community and it was where most respondents found a support network. Some of the respondents indicated that they could talk openly about their child’s disability in church, and they asked for acceptance and that attention be paid to children with disabilities and support for their carers.

*Every opportunity you have to be before people, always talk about your child in the community. Now this can be in [the] form of a testimony, if it is at church, where actually the majority of the people are the ones you meet […]. Don’t shun him away because if you don’t talk about him or her, no one else will talk about him. It’s you, if you shy away to talk about him, then the rest also will shy away to talk about him. (Father of Akiki)*

The carers were found to be crucial for awareness creation and fighting against stigma. We found that the community closest to the household, such as the church, neighbours and friends, were more accepting of the children with special needs, because they had been informed about the situation by carers. In this immediate social context, people were more familiar with disability, there were fewer misconceptions and there was greater awareness. Some carers in this study took on the role of reducing stigma. However, participants indicated that, in the wider community, for example on public transport, there were more instances where misconceptions were expressed.

*Actually what I have noticed. There are those who know about her condition and there are those who don’t know about her condition. Those who know about her condition they don’t mind, but those who don’t know, they mind so much.”* (Mother of Dembe).

*We all talk about it. You have to talk about it. If you don’t talk about it, you leave people in the dark. They will actually say that maybe it is true, it is witchcraft. We always share it in our prayer that this is not witchcraft and every time they hear it from us, we are saying it is not. Then they come to believe. Not from another person but from us.* (Mother of Akiki)

We found that a positive experience of sharing the story of their child or taking the child to church or outside the house facilitated carers and others, and they were more confident to venture outside again, which in turn reinforced social inclusion. This sets in motion the upward spiral towards visibility. We should also note that bridging social capital, such as the NGO, but also by participating in the photovoice study itself, stimulated carers to step outside and talk about their child with special needs. We found that after the photovoice study the respondents saw themselves more as advocates of children with special needs. They felt more enabled to share their story and to allow their children to participate in society.

*It is getting our children, getting children with special needs involved in the day to day activities, and to make the community where we live aware that these children also matter and are important to us. So that we deal with that stigma which sometimes affects parents and other people with children that have special needs. Because once you show the community that these children matter, they are important, they should be accepted, and they should get involved in whatever we do, they will not shy [away from] them and the stigma will be reduced, even removed.* (Father of Kingongo)

#### Downward spiral towards concealment

In contrast, a downward spiral towards concealment might also occur, which reinforces stigma. When carers did not accept the special needs of their child or feared stigmatization from outside, we found that they might be more inclined to keep their child hidden. A negative experience when talking about or trying to socially include their child might also result in the carer hiding the disability or the child even more, thus playing a significant role in shaping carers’ overall trajectory towards more exclusion.

*You know, in Uganda, it is very difficult for most of the families to accept children with special needs and it’s not easy to convince them. For sure, they don’t like those special needs children. If the parents themselves who gave birth they can ignore such children with special needs, how do you come to a conclusion that these family members will be helpful?* (Mother of Sanyu)

As a result, it was less common to see disability in the close community of these carers and their child with special needs. In the community surrounding these households, less was known about special needs and misconceptions were not challenged. This lack of awareness could in turn sustain or facilitate further stigma. This may then inhibit social inclusion, and the carer and others in the household may be less confident to venture outside the house, which further reinforces stigma and inhibits social inclusion.

Father of Muremba: *Social inclusion is very important in fighting stigma. Stigma I do not know how it is in Belgium, but here in Africa, more so in Uganda, stigma is affecting families where there are children with special needs. To an extent that some parents hide their children. Get them away from them and…*.

Mother of Akiki: *Take them in a special room in the house.*

Father of Muremba: *Yes, lock them in the bedrooms and cover them there.*

Grandmother of Abbo: *And when visitors come, they say take that one to the bedroom.*

(Focus group discussion)

*It must be an effort of every parent, or every guardian, to make sure that the child, as early as possible, should be allowed to talk, to pray, with his fellow age mates, so that it is an attitude of change, as opposed to exclude the child that can bring attitude towards others in future. So, that’s why it should start as early as possible, so that his level of social inclusion is easy, is faster.* (Father of Kingongo)

### The influence of the household on the social inclusion process

To identify the influence of the household on the social inclusion process, we will first look at the characteristics of the child with special needs and the carer who lives in the same household, before looking at the other household members. Subsequently, we will focus on the role of the household’s social network in this social inclusion process.

#### Child living with special needs

**Fig. 3:**
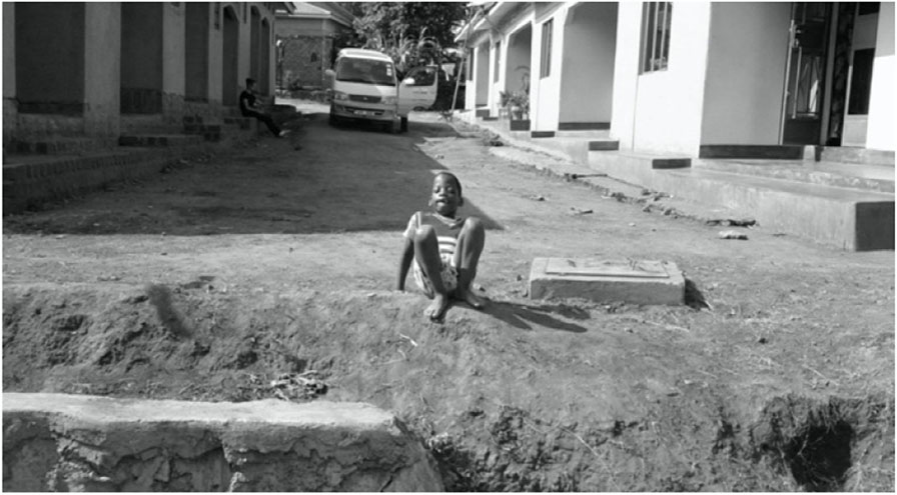
Source: Mother of Ojore

In accordance with our definition of disability, which is seen as ‘a dynamic interaction between health conditions and contextual factors, both personal and environmental’ ([14]: p.4), we found that social inclusion was limited by the fact that the surrounding environment was not adapted to children with special needs. For example, carers mentioned that public transport, such as taxis and motorcycle taxis (*boda boda*), were not adapted to accommodate children with a disability. One of the respondents mentioned that the surroundings of her house did not facilitate her son moving freely around, thus limiting the child’s inclusion – as presented in Fig. 3.

*Ojore experiences some barriers in movement due to poor sight and due to the rugged environment.* (Mother of Ojore)

Furthermore, respondents indicated that the child’s abilities and behaviour influenced the way in which they could be included. The children in this study lived with different disabilities, ranging from a physical disability to children with Down Syndrome or Cerebral Palsy, impacting what each child could do autonomously and the kind of behaviours they exhibited. Some of the children were very open and curious towards other children, which stimulated their social inclusion, while other children displayed aggressive behaviour, which negatively impacted on their social inclusion. Several respondents indicated that, as the child grew older and gained skills, there was more acceptance, both by the carers and by people outside the household, with the prejudice in their surroundings dissolving.

**Fig. 4:**
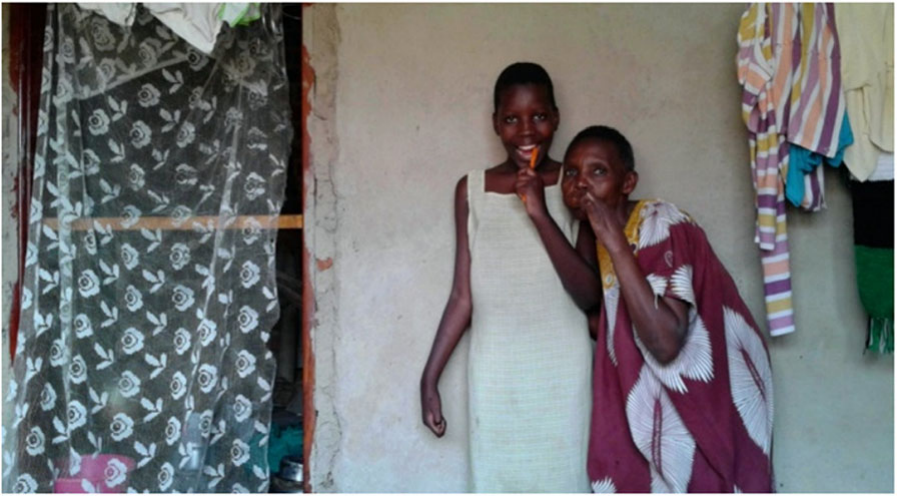
Source: Grandmother of Abbo

Related to this, the visibility of the child’s disability influenced their social inclusion in various ways. On the one hand, when a child had an obvious disability, such as missing limbs, the surrounding community responded in a more stigmatizing manner. Most carers tried to present their children in a neat and tidy manner, because they were convinced that this would stimulate their child’s social inclusion (Fig. 4). On the other hand, when a child did not display visible signs of a disability, carers mentioned that this also resulted in less understanding from others when the child behaved in an unexpected way.

*The problem is, Abbo now gets worse sometimes and she says things that don’t correspond with her age. And people look and say, that big child, why does she do those things? Yet when you look at Abbo, you cannot know that she is disabled.* (Grandmother of Abbo)

*Cleanliness – important to be in the community. (Grandmother of Abbo)*

*I am saying that we have to keep our children clean such that when we take them, let’s say to the church, they have to look clean because sometimes people can isolate them if they can have saliva and mucus on them.* (Mother of Mukisa)

#### Carer of a child living with special needs

Many of the carers were not the parents of the children. Some of the children included in this study were cared for by the extended family. There were various reasons for this, ranging from the employment of the parent abroad in a context of widespread poverty and unemployment, to a mother who did not accept the disability of her child. In several instances, only one of the parents took care of the child, due to the fact that the partner did not accept the child’s disability. Our results show that carers also go through a process of acceptance when a child with special needs is born. The respondents indicated that they first needed to overcome internal stigma and accept the disability of their child. This acceptance was necessary for them to be able to talk openly about their child’s disability and to become advocates and create awareness in the environment.

*I think it is a bit normal that every person at a certain level gets stigmatized. It is real and the stigma first starts within. For me when I gave birth to Akiki, I was the first person to stigmatize. After the doctors declared, I said: ‘How do I tell my friends?’ I kept on asking the doctor, ‘But what have I given birth to?’* (Mother of Akiki).

The carer’s self-efficacy and ownership was found to be key in the upbringing and social inclusion of their children with special needs. More specifically, they deployed innovative ways to include their child, such as inviting other children to come over to play with their toys and eat sweets, or by stimulating the development of their own child. Those carers displaying stronger ownership and self-efficacy skills also more frequently challenged the stigmatizing attitudes expressed towards their children and were more vocal in challenging the stigmatizing responses they received. The carers pointed out that they required more innovative ways of coping than with able bodied children.

*To my view, if you want to be innovative you have to involve the child who is normal and with special needs. Then you get them together in some activity that will attract these normal children to be active and play with them. That calls for us to be there because, as this one is enjoying, the other one is also participating.* (Father of Kigongo)

In contrast, we also found that a carer who lacked ownership of care for their child with special needs hampered the child’s social inclusion. In this study, one carer left the responsibility to others, resulting in the child living in very unhygienic and inhumane circumstances and reinforcing stigmatizing responses. Furthermore, the carer did not attempt to involve his social network, did not challenge the stigmatizing responses and was not open to support from an NGO. All these elements contributed to a spiral towards concealment, where the child was fully excluded from his community.

*It is only me to do each and everything. That’s why sometimes when I go for my other activities, I have to close him [in the house], not to leave him outside, because even if it rains sometimes you find that they leave him in the rain.* (Father of Gonza)

While the carers were crucial to the process of socially including the child with special needs, the presence of disability in the household also impacted on the carers’ social network. Thus, taking care of a child with special needs not only required a lot of financial and practical resources, but also had a social impact on the carers, who had less time for social activities. Their social network was often reduced to other carers of children with special needs who they met through the NGO.

#### Household in which a child with special needs lives

When discussing the household characteristics influencing the social inclusion of children with special needs, we should first note that the ‘household’ is not a uniform concept. In this study, the household ranged from a couple with children to a household headed by a grandmother to an extended family which employed help to take care of the child.

As mentioned above, the key to social inclusion is that carers themselves accept the disability of their child. This acceptance process often began within the household. While household members usually accepted the disability of the child over time, this was not always the case. In this study, one male and two female respondents explained that their partners had after many years still not come to terms with the special needs of their child.

*The father of my kids doesn’t support his children at all. Actually, he has ignored them, that he no longer cares. He doesn’t want to care about them.* (Mother of Dembe)

When all household members accept the special needs of the child, it can form a supportive context which stimulates social inclusion. Such a supportive household displays a high level of solidarity and is better equipped to develop innovative ways to integrate the child into society. However, it is not only acceptance that is a precondition to create a supportive household context. A pre-existing atmosphere of attachment and support between the household members is also required. It should be noted that a child with special needs can also have an impact on the functioning of the household. In one of the households involved in the study, the birth of two children with special needs impacted negatively on its functioning. Several respondents agreed that this social unit was key to their upbringing and social inclusion in a context where the government provided limited resources. The household members’ belief in the capabilities and value of social inclusion were key to the child’s participation in society.

*The family is very key. The family is one of the most important unit in the society. It does the grooming, the taming, it is the family to stand for you, to allow you to participate. So, to me, a family is very important in social inclusion, because it plays the basic role of allowing a child to participate. Collecting water, washing dishes, washing clothes, because all activities here, we do, they are manual activities. So, if a family doesn’t believe that a child can do something, so he will not have access to those activities. He will actually be excluded and put somewhere in a corner where they don’t want him to get out and maybe disrupt the different activities.* (Mother of Akiki)

**Fig. 5:**
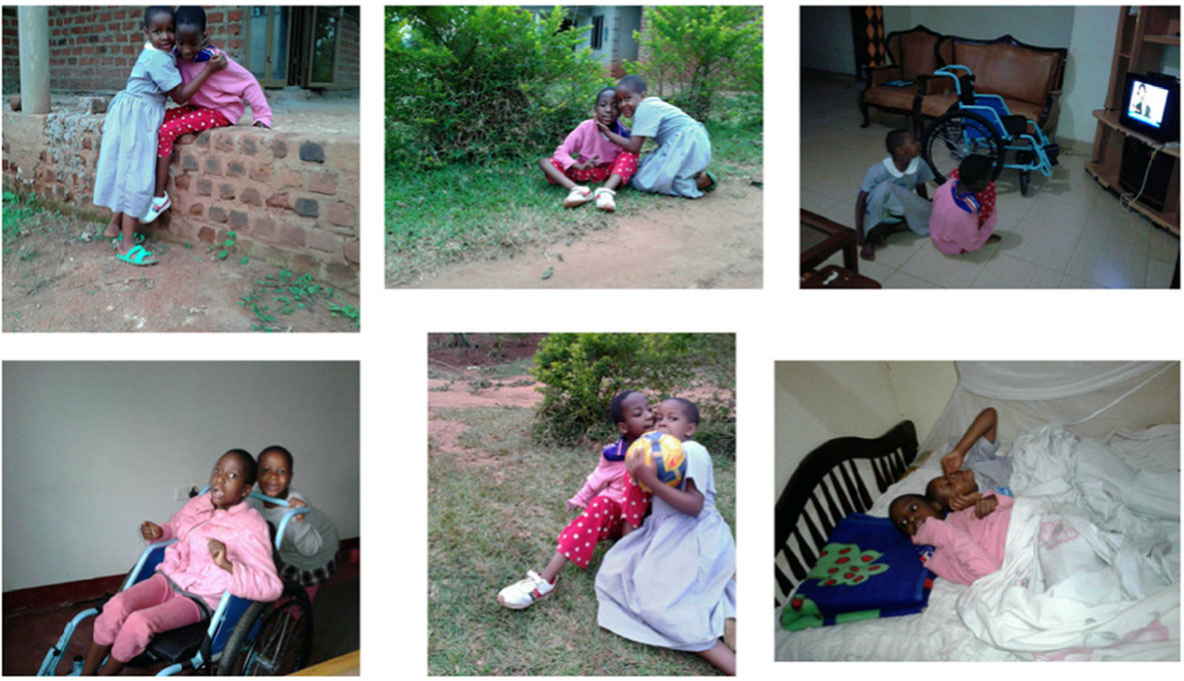
Source: Father of Muremba

When solidarity in taking care of the child is built within the household, other children in the household also became involved in care for their sibling with special needs – as shown in Fig. 5. They not only supported their brother or sister with special needs in their daily care, but also made sure they were safe in settings which were not adapted to children with special needs. They also facilitated social inclusion by involving them in games with their peers. However, sometimes caring for a sibling with special needs becomes too much. In one instance, because of the lack of involvement of the sole parent present, the younger brother who was taking care of his sibling with special needs was unable to cope and ran away from home.

*She has a little sister. The little sister gives support to Muremba and because this young sister realizes sometimes, she needs company. So she has given her sister Muremba a lot of company. Even when they are eating, they are at home, now when she was supporting her. She was going to fall from the wall, and she was there for her. When we move out, we are always moving with her, when they are seated at home, when they are watching TV, they sleep together. When she is in a wheelchair, she is driven and supported by her. So those are the photos.* (Father of Muremba)

Having a stable income means a household can contribute to the social inclusion of their children – especially in a country where there are no government facilities that provide support for children with special needs. When carers and their household members do not have the financial means, they often borrow money from friends or extended family members. Financial stability is important to buy aids, such as a wheelchair or standing frame, and medicines. Moreover, being able to pay for care from an NGO, to be able to learn, to have access to speech therapy and physiotherapy were also key to social inclusion. For example, additional finances were needed to assist one child born with only one leg and no other limbs to attend regular school. To be admitted to the school, the parents had to fulfil certain conditions.

*Condition one, he must have a wheelchair, he must have somebody to take care of him. Special for him and we pay the special person. In case he wants to go for susu, in case he wants to go for pupu, in case he, even during break time, he pushes him too … he has fallen a few times but also we have cautioned him and he has accepted … we allow other children to push him so that he mingles. He mingles with them, but …. that’s an extra cost.* (Father of Kigongo)

#### Household’s social network

It should be noted that this process of acceptance did not always start from within the household. We found that sometimes extended family members helped in this acceptance process, but not all extended family members were supportive from the start.

*Yeah the family members could not take it easy. Some were talking about Sanyu very bad and some neighbours of course were talking about him very badly. You know neighbours at times. We were not living here. Before, we were renting, but so many people were saying so many words that […] maybe we sacrificed Sanyu to get rich, which riches we don’t even have, family members were saying they had never seen such a kid in their family, so they were not supportive.* (Mother of Sanyu)

**Fig. 6:**
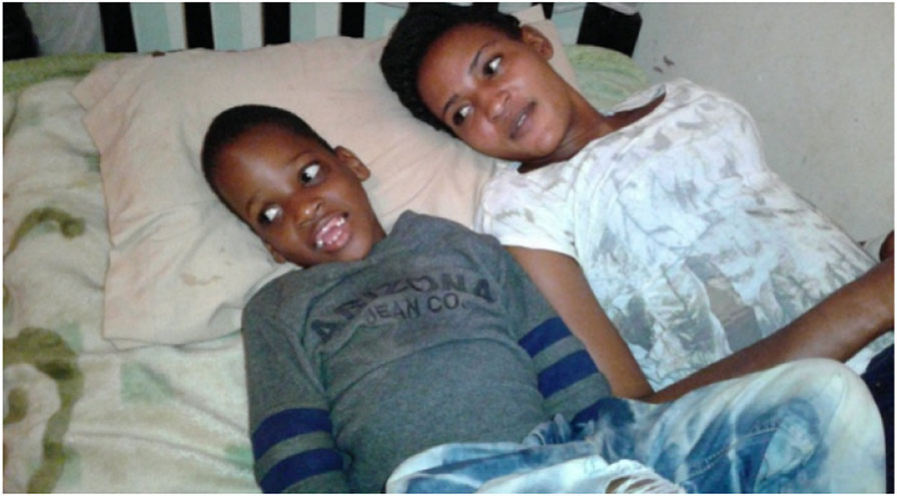
Source: Mother of Ojore

When the neighbours and extended family members accepted the child’s disability, they were found to be a source of indispensable support for the carers and their children, ranging from emotional support to support with caregiving to financial support (Fig. 6).

*He is having his dinner, being assisted still by the auntie. The auntie is always with him almost like 24 h. Like where I am not, she is next mother in line.* (Mother of Ojore)

We found that in instances where the carer was not supported by other household or extended family members, bridging social capital was crucial in supporting them in the upbringing of the child. Specialized daycare centres, which are rare in the country, were crucial to carers gaining access to the right information, educational support for their children, learning new skills and abilities and the provision of daily necessities such as food. Moreover, carers met others in the same situation and could provide emotional support to each other. We found that it also stimulated the social inclusion of the children with special needs, given that they met peers with whom they could play. Furthermore, the children were given assistance in other basic abilities, such as toilet training, and they were stimulated to improve their vocal and behavioural skills, for example to control aggressive behaviour, which indirectly improved their capacity for social inclusion. The carers indicated that more government support was needed to organize specialized care for children with special needs.

*Angel’s Centre has helped me look after Abbo. Because Abbo is taken as part of the family. And when I reached Angel’s Centre and saw other special needs children, I felt strong because I thought she was alone like her. Now Abbo also learnt how to remove her panties, she can say that ‘I want to do susu’.* (Grandmother of Abbo)

## Discussion

Approximately 13 % of Ugandan children live with some form of disability [1]. Social inclusion establishes a basis for the overall wellbeing of children with special needs. In line with the current view on disability, the focus of research has shifted towards seeing social inclusion as based on the complex interaction between personal and environmental factors, which should be better understood [2, 14]. Although children’s interpersonal environment is centred around the household, little is known about the role of the household in studies focusing on the social inclusion of children living with a disability in Uganda. Therefore, our research set out to fill this gap.

We found that many misconceptions about disability in the Ugandan community led to stigma, which was experienced by the carers and their children with special needs in various ways. Our results are in line with previous research, in which stigmatizing attitudes towards people with disabilities and their carers have been found to be entrenched in Sub-Saharan Africa communities. They ranged from being considered to be cursed to being thought of as unworthy of care to violent exclusion [15, 36]. This article showed that in such a stigmatized context, socially including a child with special needs may be highly complex because it has the potential to both benefit and cause harm. Because disability was socially devalued to a certain degree, the carers and their household members had to deal with the ongoing process of stigma management. Depending on the individual characteristics of the child, the carer and household may experience an upward spiral towards visibility or a downward spiral towards concealment, which may reinforce social inclusion or stigma, respectively. In line with Chenoweth and Stehlik (2004), social ‘inclusion is seen as a process rather than an outcome’ ([15]: p.60). In this process, carers must find a balance between social inclusion and the need to protect their children from the negative effects of stigmatization. This is in agreement with Schleien et al. (2014), who focused on parental perspectives in their study of barriers to the participation of children with special needs in recreational activities in the United States [37].

The processes described in the ‘upward spiral towards visibility’ or the ‘downward spiral towards concealment’ are reflected in the Disclosure Process Model (DPM) [38]. In this framework, Chaudoir et al. (2011) identified strategies that could assist disclosers of concealable stigmatized identities, such as mental illness or HIV, in maximizing the likelihood that disclosure will benefit their wellbeing [38]. The social inclusion of a child with special needs in the Ugandan context, where disability is highly stigmatized, might also be thought in these terms of the disclosure of a person’s concealable stigmatized identity. However, we should note that a disability is not always concealable. We found that some carers attempted to keep their child’s disability hidden by actually concealing their child. In line with DPM, socially including a child with special needs is nested within an ongoing process of stigma management, where past experiences may shape the future openness and thus the social inclusion of a child with special needs [38]. Thus, they may shape the overall social inclusion trajectory. As we found, positive experiences of inclusion may stimulate future social inclusion. However, carers who are struggling to accept the fact that their child is living with a disability and who are also living in a context in which disability is highly stigmatized, rarely have opportunities to socially include their child and are more likely to conceal them. According to Scior et al. [36], socially excluding one’s child perpetuates negative stereotypes. Corresponding to the DPM, these carers are ‘missing out on the critical verbal dialogue needed to cognitively and affectively process information’ about having a child with special needs and integrating it into the community ([38] p. 23). However, if these carers do socially include their child, they will have fewer positive experiences, which may, in turn, make them less likely to attempt to socially include their child in the future [38].

 In this study, the main drivers of inclusion of children with special needs were their carers. This is in line with Hall (2009), who also found that ‘their involvement was crucial as they provided opportunities for social inclusion’ ([7]: p. 169). Children in this study were socially included in play, household chores and church events, among other activities. Furthermore, our results add to previous research which shows that maternal self-efficacy – the carers’ perception of their own ability to handle difficulties – stimulated participation in society [9, 13]. In this study, the fathers’ and other carers’ self-efficacy also played a key role in their child’s social inclusion, broadening the findings of previous research. In line with Chenoweth and Stehlik (2004), our study also found that caring for a child with special needs negatively impacts the carers’ social network, which can lead to social isolation and less social inclusion of both the child and carer. This leads in turn to limited access to external support and a need to rely on internal resources to meet care needs [15].

In addition to the characteristics of the carer, the household also influenced the upward or downward spiral towards social inclusion or exclusion, respectively. Household stability, such as a supportive environment among household members, the presence of adequate financial resources and access to information all contributed to the social inclusion of the children with special needs. This was in line with Richard (2014) [11]. More specifically, lack of financial means were found to inhibit access to bridging social capital, and thus hampered social inclusion of the child [11, 13]. In turn, caring for a child with special needs added to the financial burden carried by the household, which was ‘internally focused on the task of survival with no “surplus” resources to contribute to the building of social capital’ ([15]: p. 66). Furthermore, our results were in line with previous research which found that a household’s inclusion in the extended family and community networks such as the church and NGO positively influenced the child’s participation in society [11, 15].

This study also has several limitations. First, a selection bias should be noted, as the carers who participated in our photovoice research project were more likely to be positively involved in the care of their child than less or non-involved carers. Nevertheless, one respondent who took part in the study fell into the latter category. Furthermore, the participants were part of a parental support group at a local NGO. The fact that these children already accessed daycare and the parents were involved in the NGO might present a further selection bias. Future research should thus include carers and children with special needs who are not accessing any form of care. Second, in this study, social inclusion was mainly operationalized as involvement in activities. This was in line with the carers’ understanding of the concept of social inclusion. By providing an insight into the perspective of the carers on the social inclusion of their children, this article responded to a research need expressed in a recent review study by Koller et al. (2017), who noted that ‘to date, no studies have specifically focused on how parents understand social inclusion for their children with disabilities, besides recognizing the essential value of peer relationships for their children’s development and well-being’ ([9]: p. 5). A family’s understanding of the social inclusion of a child with a disability in turn affects ‘the child’s involvement in family life, parents’ expectations and resource allocations, and the child’s access to development and growth opportunities’ ([11]: p. 309). However, future studies should also involve the perspective of the children themselves, discussing a broader understanding of social inclusion. Hall (2009) identified three elements which are key to the social inclusion of people living with a disability: ‘involvement in activities, maintaining reciprocal relationships, and a sense of belonging’ ([7]: p 171). Finally, longitudinal qualitative research might be undertaken to account for the dynamics of household boundaries and composition, both of which change over time. The household as an entity represents but a moment in the dynamic process of its continual formation and reformation [39–45].

One of the strengths of this study is the use of the photovoice methodology, which responded to a need for research in which families can ‘represent themselves in ways that enable them to share their own points-of-view, issues of concern, or creative insights’ ([29]: p.1). This led the carers to perceive themselves as advocates for their children after the study, which is in line with previous research [31]. The carers felt more encouraged and better able to share their stories after the photovoice study. Furthermore, their participation in the photovoice research project seemed to positively encourage them to speak more openly about their children and thus indirectly stimulated their children’s social inclusion. However, this methodological effect must be further examined in future research.

This research is both relevant for academic and policy purposes. With respect to both, this study has the potential to plug a gap in the understanding of the social inclusion of children with a disability in resource-limited settings. Despite the fact that disability is prevalent among Ugandan children, it remains a ‘hidden reality’ [46]. This article takes a new step in laying a foundation for the establishment of sound policies, by drawing attention to this ‘hidden reality’ and to the life situation of children with disabilities in Uganda [46]. This study supports Carter’s (2013) claim that ‘a logical tactic in the ongoing battle against stigma is social inclusion’ ([47]: p.773). Based on the research results, which showed that awareness creation, stigma reduction and social inclusion all go hand in hand, this article supports the need for local governments and other stakeholders to become involved in fighting misconceptions about disability and creating more awareness. This could be done through advocacy efforts and public awareness campaigns, among other strategies [47]. In addition, government should provide access to specialized care and support for children with special needs and their households. This awareness creation, as well as social and medical support, is crucial to assisting households to form a supportive environment for their child with special needs, which will ultimately promote the children’s social inclusion and wellbeing [11].

## Conclusions

Many misconceptions about disability in the Ugandan community studied lead to stigma, ranging from the children and parents being cursed to children not being considered worthy of care to violent exclusion. In such a context where disability is highly socially devalued, the social inclusion of children with special needs may be very complex because it has the potential to both benefit and cause harm. Our results showed that in such a context, carers and other household members must deal with an ongoing process of stigma management. Influenced by the individual characteristics of the child (i.e. their abilities and behaviour; surroundings not adapted to the child’s specific needs), the carer (i.e. acceptance, self-efficacy and ownership) and the household (i.e. context of solidarity, attachment and support; financial stability; social network), the social inclusion of a child with special needs can move in an upward spiral towards more visibility or a downward spiral towards concealment that might reinforce social inclusion or stigma, respectively.

## Data Availability

The data supporting the conclusions of this article is available upon reasonable request from the authors.

## Footnotes

1 While some studies use the terms family and household interchangeably, others do not regard these concepts as synonymous *(*[18]*. National Department of Social Development, White paper on families in South Africa. 2012: Pretoria, *[19]*. Manciaux, M.R.G. and M.A. Belsey, Family Health, Research Policy and Cooperation, Editor. 2004, WHO: Geneva, *[20]*. Niehof, A., A micro-ecological approach to home care for AIDS patients. Medische Antropologie, 2004.****16****(2): p. 245–265.*). There are a myriad of definitions of the household or family. In this study, ‘household’ is defined as a ‘co-residential unit, usually family-based in some way, which takes care of resource management and primary needs of its members’ *(*[21]*. Niehof, A., Conceptualizing the household as an object of study. International Journal of Consumer Studies, 2011.****35****(5): p. 488–497.).* We use the term ‘family’ when referring to the extended family, who are not members of the household.
